# The relationship between computed tomography‐derived body composition, systemic inflammatory response, and survival in patients undergoing surgery for colorectal cancer

**DOI:** 10.1002/jcsm.12357

**Published:** 2018-11-20

**Authors:** Ross D. Dolan, Arwa S. Almasaudi, Ly B. Dieu, Paul G. Horgan, Stephen T. McSorley, Donald C. McMillan

**Affiliations:** ^1^ Academic Unit of Surgery University of Glasgow Glasgow United Kingdom

**Keywords:** Colorectal cancer, TNM stage, Systemic inflammation, Glasgow prognostic score, Body composition, Computed tomography

## Abstract

**Introduction:**

Colorectal cancer is the fourth leading cause of cancer mortality in developed countries. There is evidence supporting a disproportionate loss of skeletal muscle as an independent prognostic factor. The importance of the systemic inflammatory response as a unifying mechanism for specific loss of skeletal muscle mass in patients with cancer is increasingly recognized. The aim of the present study was to delineate the relationship between the systemic inflammatory response, skeletal muscle index (SMI), skeletal muscle density (SMD), and overall survival in patients with colorectal cancer.

**Materials and methods:**

The study included 650 patients with primary operable colorectal cancer. Computed tomography scans were used to define the presence of visceral obesity, sarcopenia (low SMI), and myosteatosis (low SMD). Tumour and patient characteristics were recorded. Survival analysis was carried out using univariate and multivariate Cox regression.

**Results:**

A total of 650 patients (354 men and 296 women) were included. The majority of patients were over 65 years of age (64%) and overweight or obese (68%). On univariate survival analysis, age, ASA, TNM stage, modified Glasgow Prognostic Score (mGPS), body mass index, subcutaneous fat index, visceral obesity, SMI, and SMD were significantly associated with overall survival (all *P* < 0.05). A low SMI and SMD were significantly associated with an elevated mGPS (<0.05). On multivariate analysis, SMI (Martin) [hazard ratio (HR) 1.50, 95% confidence interval (CI) 1.04–2.18, *P* = 0.031], SMD (Xiao) (HR 1.42, 95% CI 0.98–2.05, *P* = 0.061), and mGPS (HR 1.44, 95% CI 1.15–1.79, *P* = 0.001) were independently associated with overall survival. SMD but not SMI was significantly associated with ASA (*P* < 0.001).

**Conclusions:**

This study delineates the relationship between the loss of quantity and quality of skeletal muscle mass, the systemic inflammatory response, and survival in patients with operable colorectal cancer.

## Introduction

Colorectal cancer (CRC) is the fourth leading cause of cancer mortality in developed countries.^1^ Despite death rates from CRC falling by ~14% over the last decade, ~40% of those diagnosed will die from their cancer. Similar to most common solid tumours, disease progression is associated with a progressive nutritional and functional decline resulting in poor response to treatment and poor survival.^2^, ^3^

In the past, weight loss and body mass index (BMI) have been used as an indicator of such nutritional decline and poor prognosis.^2^, ^3^ However, because of the increased number of patients presenting in an overweight or obese state in the developed world, the use of simple weight loss and BMI as a prognostic indicator has been questioned.^4^, ^5^, ^6^, ^7^ The ability to use routine computed tomography (CT) scans to measure body composition, in particular skeletal muscle, has resulted in a marked increase in interest in using skeletal muscle index (SMI) and skeletal muscle density (SMD) to predict outcomes in patients with cancer, particularly in CRC.^8^

There is evidence supporting a disproportionate loss of skeletal muscle tissue to be an independent prognostic factor for both cancer specific and overall survival in patients with CRC.^9^ Specifically, muscle loss has been associated with poor treatment tolerance and efficacy,^10^ worse quality of life, and increased morbidity.^11^ For example, in a large study, Caan *et al*. reported that in patients with CRC, there was a significant association between lower SMI and worse overall survival.^12^ Also, Malietzis *et al*. reported that in patients with CRC, there was a significant association between lower SMD and worse overall survival.^13^

The importance of the systemic inflammatory response as a unifying mechanism for weight loss and loss of lean tissue in patients with cancer is increasingly recognized.^3^, ^14^, ^15^ Therefore, it is of interest that SMI and SMD have been repeatedly reported to be inversely associated with measures of the systemic inflammatory response, such as the neutrophil lymphocyte ratio (NLR) and modified Glasgow prognostic score (mGPS),^16^, ^17^, ^18^, ^19^, ^20^, ^21^, ^22^ that are recognized to have prognostic value in their own right.^23^, ^24^ However, this relationship is not clear. It is possible that some patients with sarcopenia may have systemic inflammation and some patients with myosteatosis might similarly have systemic inflammation, but the coexistence of those three features is poorly understood. If the above association was due to the erosion of the SMI and SMD by an ongoing systemic inflammatory response, it might be anticipated that the prognostic value of SMI and SMD was largely dependent on the presence of a systemic inflammatory response. It might also be anticipated that low SMI and SMD would influence the relationship between the systemic inflammatory response and survival.

To our knowledge, no study has comprehensively examined the relationship between CT‐derived body composition, systemic inflammatory response, as measured by the mGPS, and survival in patients with primary operable CRC. Therefore, the aim of the present study was to examine the above relationships in a prospectively maintained database of patients with CRC undergoing potentially curative resection.

## Materials and methods

### Patients

Consecutive patients who underwent elective, potentially curative resection for CRC between March 2008 and June 2017 at a single centre were identified from a prospectively maintained database. Those patients with a pre‐operative CT scan and a recorded height and weight were included.

Patients were classified according to BMI as underweight (BMI < 18.5), normal weight (BMI 18.5–24.9), overweight (BMI 25.0–29.9), and obese (BMI ≥ 30) and were recorded. All tumours were staged according to TNM fifth edition. Pre‐operative haematological and biochemical markers were recorded.

The cause and date of death were confirmed with the Registrar General (Scotland) until 1 June 2017 that served as the censor date. Informed consent was obtained from patients prior to surgery. Those with metastatic CRC and those who underwent emergency surgery or palliative surgery were excluded from the study. Ethical approval was granted by the West of Scotland Research Ethics Committee, Glasgow.

### Methods

Computed tomography images were obtained at the level of the third lumbar vertebra as previously described.^16^ Patients whose scans were taken 3 months or more prior to their surgery were excluded from the study. Scans with significant movement artefact or missing region of interest were not considered for inclusion. Each image was analysed using a free‐ware programme (NIH ImageJ version 1.47, http://rsbweb.nih.gov/ij/) shown to provide reliable measurements.^22^

Region of interest measurements were made of visceral fat area (VFA), subcutaneous fat area, and skeletal muscle area (cm^2^) using standard Hounsfield unit (HU) ranges (adipose tissue −190 to −30 and skeletal muscle −29 to +150). These were then normalized for height^2^ to create indices: subcutaneous fat index (SFI, cm^2^/m^2^) and SMI (cm^2^/m^2^). Skeletal muscle radiodensity (SMD, HU) was measured from the same region of interest used to calculate SMI, as its mean HU.

Visceral obesity was defined as VFA > 160 cm^2^ for male patients and >80 cm^2^ for female patients.^25^ Sarcopenia was defined as described by Martin *et al*. as an SMI < 43 cm^2^/m^2^ if BMI < 25 kg/m^2^ and SMI < 53 cm^2^/m^2^ if BMI ≥ 25 kg/m^2^ in male patients and an SMI < 41 cm^2^/m^2^ in female patients.^6^ Sarcopenia was also described by Caan *et al*. as an SMI < 52.3 cm^2^/m^2^ if BMI < 30 kg/m^2^ and SMI < 54.3 cm^2^/m^2^ if BMI ≥ 30 kg/m^2^ in male patients and an SMI < 38.6 cm^2^/m^2^ if BMI < 30 kg/m^2^ and SMI < 46.6 cm^2^/m^2^ if BMI ≥ 30 kg/m^2^ in female patients.^12^ Myosteatosis was defined by Martin *et al*. as an SMD < 41 HU in patients with BMI < 25 kg/m^2^ and <33 HU in patients with BMI > 25 kg/m^2^.^6^ Myosteatosis was also defined by Xiao *et al*. as <35.5 HU in men and <32.5 HU in women.^26^ Subcutaneous fat index was defined as ≥50.0 cm^2^/m^2^ in men and ≥42.0 cm^2^/m^2^ in women^27^ (*Table* 1).

**Table 1:** Computed tomography‐derived body composition measures and thresholds used

**Body Composition Measurement**	**Frequency n (%)**
**High SFI** ^**27**^ **:**	
Males>50.0 cm^2^m^2^ and Females>42.0 cm^2^m^2^	No: 116 (17.8%) Yes: 534 (82.2%)
**Visceral obesity** ^**5,6**^ **:**	
VFA: Males >160 cm2 and Females >80 cm2	No: 177 (27.2%) Yes: 473 (72.8%)
**Sarcopenia**	
**SMI (Martin)** ^**6**^ **:**	
Males: BMI < 25 kg/m^2^ and SMI < 43 cm^2^ m^2^ or BMI ≥ 25 kg/m^2^ and SMI < 53 cm^2^ m^2^ Females: BMI < 25 kg/m^2^ and SMI < 41 cm^2^ m^2^ or BMI ≥ 25 kg/m^2^ and SMI < 41 cm^2^ m^2^	No: 367 (56.5%) Yes: 283 (43.5%)
**SMI (Dolan BMI ≥ 25):** Males: BMI < 25 kg/m^2^ and SMI < 45 cm^2^ m^2^ or BMI ≥ 25 kg/m^2^ and SMI < 53 cm^2^ m^2^ Females: BMI < 25 kg/m^2^ and SMI < 39 cm^2^ m^2^ or BMI ≥ 25 kg/m^2^ and SMI < 41 cm^2^ m^2^	No: 371 (57.1%) Yes: 279 (42.9%)
**SMI (Caan)** ^**12**^ **:**	
Males: BMI < 30 kg/m^2^ and SMI < 52.3 cm^2^ m^2^ or BMI ≥ 30 kg/m^2^ and SMI < 54.3 cm^2^ m^2^ Females: BMI < 30 kg/m^2^ and SMI < 38.6 cm^2^ m^2^ or BMI ≥ 30 kg/m^2^ and SMI < 46.6 cm^2^ m^2^	No: 313 (48.2%) Yes: 337 (51.8%)
**SMI (Dolan BMI ≥ 30)**	
Males: BMI < 30 kg/m^2^ and SMI < 45.6 cm^2^ m^2^ or BMI ≥ 30 kg/m^2^ and SMI < 56.8 cm^2^ m^2^ Females: BMI < 30 kg/m^2^ and SMI < 39.1 cm^2^ m^2^ or BMI ≥ 30 kg/m^2^ and SMI < 44.6 cm^2^ m^2^	No: 386 (59.4%) Yes: 264 (40.6%)
**Myosteatosis**	
**SMD (Martin)** ^**6**^ **:**	
BMI < 25 kg/m^2^ and SMD < 41 HU or BMI ≥ 25 kg/m^2^ and SMD < 33HU	No: 258 (39.7%) Yes: 392 (60.3%)
**SMD (Dolan BMI ≥ 25)**	
BMI < 25 kg/m^2^ and SMD < 34 HU or BMI ≥ 25 kg/m^2^ and SMD < 32HU	No: 343 (52.8%) Yes: 307 (47.2%)
**SMD (Xiao)** ^**26**^ **:**	
Males<35.5HU and Females<32.5HU	No: 309 (47.5%) Yes: 341 (52.5%)
**SMD (Dolan Male/Female)**	
Males<34.1 HU and Females<HU 34.4 HU	No: 304 (46.8%) Yes: 346 (53.2%)

BMI, body mass index; SFI, subcutaneous fat index; SMD, skeletal muscle density; SMI, skeletal muscle index; VFA, visceral fat area.

Measurements were performed by two individuals (A. S. A. and L. B. D.), and inter‐rater reliability was assessed in a sample of 30 patient images using inter‐class correlation coefficients (ICCCs) (total fat area ICCC = 1.000; subcutaneous fat area ICCC = 1.000; VFA ICCC = 1.000; skeletal muscle area ICCC = 0.998; and SMD ICCC = 0.972). Investigators were blind to patient's demographic and clinicopathological status.

An autoanalyser was used to measure serum C‐reactive protein (mg/L) and albumin (g/L) concentrations (Architect; Abbot Diagnostics, Maidenhead, UK). The mGPS, NLR, and neutrophil‐platelet score were derived as previously described.^28^

### Statistical analysis

Body composition measurements were presented as median and range and compared using Mann–Whitney or Kruskal–Wallis tests. Categorical variables were analysed using χ^2^ test for linear‐by‐linear association or χ^2^ test for two‐by‐two tables.

Mortality within 30 days of the index procedure or during the index admission was excluded from subsequent survival analysis. The time between the date of surgery and the date of death of any cause was used to define overall survival. Survival data were analysed using univariate and multivariate Cox regression. Those variables associated with a degree of *P* < 0.1 were entered into a backward conditional multivariate model.

Missing data were excluded from analysis on a variable‐by‐variable basis. Two‐tailed *P*‐values < 0.05 were considered statistically significant. Statistical analysis was performed using SPSS software (version 21.0; SPSS Inc., Chicago, IL, USA).

## Results

In the present study, although ImageJ software was used to calculate body composition parameters, the SMI and SMD threshold values used were from the Martin and Caan groups who both used Slice‐O‐Matic software.^6^ However, Richards *et al*. compared Slice‐O‐Matic and ImageJ calculated results in 174 patients with primary operable CRC with an ICCC of 0.953 (*P* < 0.01).^16^ Therefore, the use of ImageJ software was unlikely to introduce a large error unto the present results. Indeed, the use of such open source software is likely to facilitate comparison of studies across different cancer types and research institutions.

In total, 832 patients were identified as having undergone potentially curative surgery for CRC. Of these, 182 were excluded because of missing eligible CT scans, clinicopathological data, or blood test results. A further five patients were excluded as they died in the immediate post‐operative period. A total of 650 patients (354 men and 296 women) were included in the final analyses.

There have been a number of definitions of SMI using CT scans. Nevertheless, it is clear that muscle mass varies in male and female patients and with BMI. Skeletal muscle index has been defined differently in male and female patients and according to BMI, which are summarized in *Table* 1. In the present study, SMI (Dolan) thresholds were derived using receiver operating characteristic curve analysis to determine thresholds associated with overall survival in this population. This was also conducted using validated online biomarker cut‐off optimization software.^29^ In male patients, the clinically significant cut‐off for SMI with a BMI < 25 was 45 cm^2^/m^2^ and for male patients with a BMI ≥ 25 was 53 cm^2^/m^2^. The clinically significant cut‐off for SMI in female patients with a BMI < 25 was 39 cm^2^/m^2^ and for female patients with a BMI ≥ 25 was 41 cm^2^/m^2^. Given that these SMI threshold values (Dolan BMI ≥ 25) were similar to those of Martin (*Table* 1) and to facilitate comparison of studies, the threshold values of Martin were used in the analysis. In addition, the association between sarcopenia (Martin) and sarcopenia (Dolan BMI ≥ 25) was strong (*P* < 0.001). For example, when Martin *et al*. thresholds were used, 43.5% of patients had sarcopenia, and when Dolan *et al*. thresholds were used, 42.9% of patients had sarcopenia (*Table* 1).

In the present study in male patients, the clinically significant cut‐off for SMI with a BMI < 30 was 45.6 cm^2^/m^2^ and for male patients with a BMI ≥ 30 was 56.8 cm^2^/m^2^. The clinically significant cut‐off for SMI in female patients with a BMI < 30 was 39.1 cm^2^/m^2^ and for female patients with a BMI ≥ 30 was 44.6 cm^2^/m^2^. Given that these SMI threshold values (Dolan BMI ≥ 30) were not similar to those of Caan (*Table* 1), the threshold values of Caan were not used in the subsequent analysis.

With reference to SMD, Martin *et al*. in 1473 patients with multistage lung and gastrointestinal cancers defined SMD (myosteatosis) as an SMD < 41 HU in patients with BMI < 25 kg/m^2^ and <33 HU in patients with BMI ≥ 25 kg/m.^6^ In contrast, Xiao *et al*. in 3051 non‐metastatic stage I–III CRC defined myosteatosis according to sex as <35.5 HU in men and <32.5 HU in women.^26^ In the present study, SMD (Dolan) thresholds were derived using receiver operating characteristic curve analysis to determine thresholds associated with overall survival in this population. This was also conducted using validated online biomarker cut‐off optimization software.^29^ The clinically significant cut‐off for SMD in patients in the present cohort with a BMI < 25 was 34 HU and for patients with a BMI ≥ 25 was 32 HU. Given that these SMD threshold values (Dolan BMI ≥ 25) were not similar to those of Martin, the threshold values of Martin were not used in the subsequent analysis.

In the present study, the clinically significant cut‐off for SMD in male patients was 34.1 HU and in female patients was 34.4 HU. Given that these SMD threshold values (Dolan Male/Female) were similar to Xiao and to facilitate comparison of studies, the threshold values of Xiao were used in the analysis. In addition, the association between SMD (Xiao) and SMD (Dolan Male/Female) was strong (*P* < 0.001). For example, when Xiao *et al*. thresholds were used, 47.5% of patients had myosteatosis, and when Dolan *et al*. thresholds were used, 46.8% of patients had myosteatosis.

**Figure 1:**
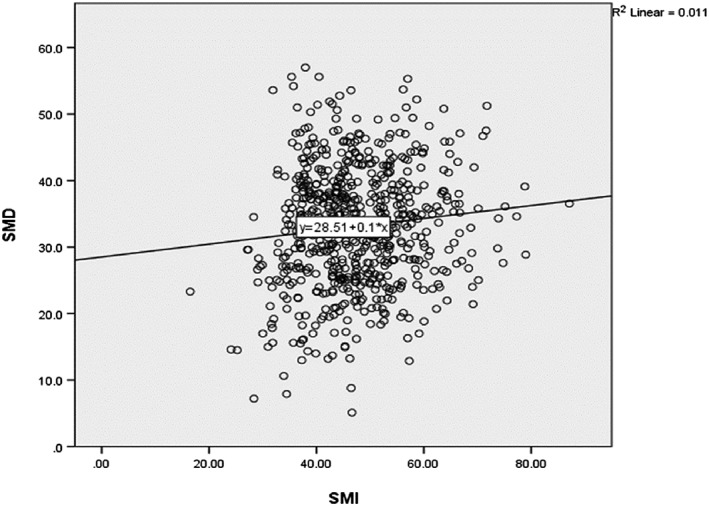
The relationship between skeletal muscle index (SMI) and skeletal muscle density (SMD) in patients undergoing elective surgery for colorectal cancer (*n* = 650).

The relationship between clinicopathological characteristics, body composition, and overall survival is shown in *Table* 2. The majority of patients were over 65 years of age (64%), overweight or obese (68%), with some co‐morbidities (88%) and node negative disease (67%). The majority of tumours were located in the right colon (38%) and rectum (37%), and an open surgical approach was applied in 62% of cases. A total of 528 patients were alive at the censor date with a median survival of 44 months (range 1–110 months). Deaths by any cause occurred in 122 patients (18%), 71 (11%) of which were cancer specific. On univariate survival analysis, age, ASA, TNM stage, and mGPS were significantly associated with overall survival (all *P* < 0.001). Of the body composition parameters, BMI, SFI, VO, SMI (Martin, Dolan, and Caan), and SMD (Martin, Dolan, and Xiao) were significantly associated with overall survival (all *P* < 0.05). Skeletal muscle index and SMD were weakly associated (*Figure* 1). Comparing SMI (Martin) and SMD (Xiao), both SMI (HR 1.68, 95% CI 1.17–2.41, *P* = 0.005) and SMD (HR 1.47, 95% CI 1.02–2.11, *P* = 0.040) were independently associated with overall survival.

**Table 2:** The relationship between clinicopathological characteristics, computed tomography‐derived body composition, and survival in patients undergoing elective surgery for colorectal cancer (*n* = 650): univariate survival analysis

Characteristic		*n* = 650 (%)	Overall survival HR (95% CI)	*P*‐value
	Clinicopathological			
Age	≤65	234 (36.0)	1.64 (1.29–2.08)	<0.001
	65–74	251 (38.6)		
	>74	165 (25.4)		
Sex	Female	296 (45.5)	1.19 (0.83–1.70)	0.351
	Male	354 (54.5)		
ASA score	1	141 (21.7)	1.56 (1.23–1.97)	<0.001
	2	297 (45.7)		
	3	193 (29.7)		
	4	19 (2.9)		
Laparoscopic surgery	No	407 (62.6)	0.68 (0.45–1.03)	0.072
	Yes	243 (37.4)		
TNM	0	14 (2.2)	1.67 (1.31–2.14)	<0.001
	I	155 (23.8)		
	II	263 (40.5)		
	III	218 (33.5)		
Venous invasion	No	266 (40.9)	1.26 (0.87–1.82)	0.217
	Yes	384 (59.1)		
Tumour location	Right and transverse	247 (38.0)	0.84 (0.58–1.23)	0.373
	Left	145 (22.3)		
	Rectum	237 (36.5)		
	Total and subtotal	21 (3.2)		
Adjuvant chemotherapy	No	463 (71.2)	0.70 (0.45–1.08)	0.102
	Yes	187 (28.8)		
	Systemic inflammation			
mGPS	0	499 (76.8)	1.55 (1.25–1.91)	<0.001
	1	63 (9.7)		
	2	88 (13.5)		
NLR	≤3	369 (56.8)	1.40 (0.98–1.99)	0.066
	>3	281 (43.2)		
NPS	0	568 (87.4)	1.66 (1.16–2.36)	0.005
	1	67 (10.3)		
	2	15 (2.3)		
	Body composition			
BMI (kg/m^2^)	<25	29 (4.5)	0.60 (0.39–0.91)	0.0154
	≥25	190 (29.2)		
High SFI	No	116 (17.8)	0.60 (0.40–0.89)	0.011
	Yes	534 (82.2)		
Visceral obesity	No	177 (27.2)	0.68 (0.47–0.98)	0.040
	Yes	473 (72.8)		
Low SMI (sarcopenia)				
SMI (Martin)	No	367 (56.5)	1.74 (1.21–2.49)	0.003
	Yes	283 (43.5)		
SMI (Dolan BMI ≥ 25)	No	371 (57.1)	1.77 (1.24–1.54)	0.002
	Yes	279 (42.9)		
SMI (Caan)	No	313 (48.2)	1.58 (1.09–2.28)	0.016
	Yes	337 (51.8)		
SMI (Dolan BMI ≥ 30)	No	386 (59.4)	1.60 (1.12–2.28)	0.010
	Yes	264 (40.6)		
Low SMD (myosteatosis)				
SMD (Martin)	No	258 (39.7)	1.84 (1.25–2.72)	0.002
	Yes	392 (60.3)		
SMD (Dolan BMI ≥ 25)	No	343 (52.8)	1.57 (1.10–2.25)	0.013
	Yes	307 (47.2)		
SMD (Xiao)	No	309 (47.5)	1.54 (1.07–2.22)	0.020
	Yes	341 (52.5)		
SMD (Dolan Male/Female)	No	304 (46.8)	1.58 (1.10–2.27)	0.014
	Yes	346 (53.2)		

BMI, body mass index; CI, confidence interval; HR, hazard ratio; mGPS, modified Glasgow prognostic score; NLR, neutrophil lymphocyte ratio; NPS, neutrophil‐platelet score; SFI, subcutaneous fat index; SMD, skeletal muscle density; SMI, skeletal muscle index.

The relationship between SMI (Martin), SMD (Xiao), and mGPS and the clinicopathological characteristics is shown in *Tables* 3, 4, 5, respectively. A low SMI (Martin) was significantly associated with older age, higher mGPS, lower BMI, and lower SMD (Martin, Dolan, and Xiao) (all *P* < 0.001). A low SMD (Xiao) was significantly associated with older age, female sex, higher ASA a right‐sided tumour, mGPS, lower BMI, SFI, VO, and lower SMI (Martin, Dolan, and Xiao) (all *P* < 0.05). An elevated mGPS was significantly associated with a high ASA, TNM stage, tumour location, NLR, neutrophil‐platelet score, BMI ≥ 25, SMI (Martin, Dolan, and Caan), and SMD (Martin and Dolan) (all *P* < 0.05).

**Table 3:** The relationship between sarcopenia (Martin), clinicopathological characteristics, and systemic inflammation in patients undergoing elective surgery for colorectal cancer (*n* = 650)

Characteristic		High SMI (no sarcopenia *n* = 367)	Low SMI (sarcopenia *n* = 283)	*P*‐value
	Clinicopathological			
Age	≤65	160 (43.6)	74 (26.1)	<0.001
	65–74	133 (36.2)	118 (41.7)	
	>74	74 (20.2)	91 (32.2)	
Sex	Female	163 (44.4)	133 (47.0)	0.513
	Male	204 (55.6)	150 (53.0)	
ASA score	1	81 (22.1)	60 (21.2)	0.159
	2	167 (45.5)	130 (45.9)	
	3	113 (30.8)	80 (28.3)	
	4	6 (1.6)	13 (4.6)	
Laparoscopic surgery	No	220 (59.9)	187 (66.1)	0.109
	Yes	147 (40.1)	96 (33.9)	
TNM	0	9 (2.5)	5 (1.8)	0.032
	I	101 (27.5)	54 (19.1)	
	II	133 (36.2)	130 (45.9)	
	III	124 (33.8)	94 (33.2)	
Venous invasion	No	154 (42.0)	112 (39.6)	0.540
	Yes	213 (58.0)	171 (60.4)	
Tumour location	Right and transverse	138 (37.6)	109 (38.5)	0.293
	Left	77 (21.0)	68 (24.0)	
	Rectum	143 (39.0)	94 (33.2)	
	Total and subtotal	9 (2.5)	12 (4.2)	
Adjuvant chemotherapy	No	208 (56.7)	177 (62.5)	0.091
	Yes	159 (43.3)	106 (37.5)	
	Systemic inflammation			
mGPS	0	298 (81.2)	201 (71.0)	<0.001
	1	39 (10.6)	24 (8.5)	
	2	30 (8.2)	58 (20.5)	
NLR	≤3	220 (59.9)	149 (52.7)	0.063
	>3	147 (40.1)	134 (47.3)	
NPS	0	328 (89.4)	240 (84.8)	0.220
	1	32 (8.7)	35 (12.4)	
	2	7 (1.9)	8 (2.8)	
	Body composition			
BMI (kg/m^2^)	<25	103 (28.1)	116 (41)	0.001
	≥25	264 (71.9)	167 (59)	
High SFI	No	67 (18.3)	49 (17.3)	0.756
	Yes	300 (81.7)	234 (82.7)	
Visceral obesity	No	98 (26.7)	79 (27.9)	0.731
	Yes	269 (73.3)	204 (72.1)	
Low SMI (sarcopenia)				
SMI (Dolan BMI ≥ 25)	No	356 (97.0)	15 (5.3)	<0.001
	Yes	11 (3.0)	268 (94.7)	
SMI (Caan)	No	275 (74.9)	38 (13.4)	<0.001
	Yes	92 (25.1)	245 (86.6)	
SMI (Dolan BMI ≥ 30)	No	315 (85.8)	71 (25.1)	<0.001
	Yes	52 (14.2)	212 (74.9)	
Low SMD (myosteatosis)				
SMD (Martin)	No	177 (48.2)	81 (28.6)	<0.001
	Yes	190 (51.8)	202 (71.4)	
SMD (Dolan BMI ≥ 25)	No	224 (61.0)	119 (42.0)	<0.001
	Yes	143 (39.0)	164 (58.0)	
SMD (Xiao)	No	196 (53.4)	113 (39.9)	0.001
	Yes	171 (46.6)	170 (60.1)	
SMD (Dolan BMI Male/Female)	No	197 (53.7)	107 (37.8)	<0.001
	Yes	170 (46.3)	176 (62.2)	

BMI, body mass index; mGPS, modified Glasgow prognostic score; NLR, neutrophil lymphocyte ratio; NPS, neutrophil‐platelet score; SFI, subcutaneous fat index; SMD, skeletal muscle density; SMI, skeletal muscle index.

**Table 4:** The relationship between SMD (Xiao), clinicopathological characteristics, and systemic inflammation in patients undergoing surgery for colorectal cancer (*n* = 650)

Characteristic		Low SMD (Xiao)		
		No (*n* = 309)	Yes (*n* = 341)	*P*‐value
	Clinicopathological			
Age	≤65	149 (48.2)	85 (24.9)	<0.001
	65–74	108 (35.0)	143 (41.9)	
	>75	52 (16.8)	113 (33.1)	
Sex	Female	167 (54.0)	129 (37.8)	<0.001
	Male	142 (46.0)	212 (62.2)	
ASA score	1	91 (29.4)	50 (14.7)	<0.001
	2	140 (45.3)	157 (46.0)	
	3	72 (23.3)	121 (35.5)	
	4	6 (1.9)	13 (3.8)	
Laparoscopic surgery	No	195 (63.1)	212 (62.2)	0.805
	Yes	114 (36.9)	129 (37.8)	
TNM	0	7 (2.3)	7 (2.1)	0.934
	I	77 (24.9)	78 (22.9)	
	II	123 (39.8)	140 (41.1)	
	III	102 (33.0)	116 (34.0)	
T stage	0	7 (2.3)	7 (2.1)	0.327
	1	34 (11.0)	45 (13.2)	
	2	59 (19.1)	45 (13.2)	
	3	160 (51.8)	184 (54.0)	
	4	49 (15.9)	60 (17.6)	
N stage	0	208 (67.3)	226 (66.3)	0.898
	1	76 (24.6)	84 (24.6)	
	2	25 (8.1)	31 (9.1)	
Venous invasion	No	133 (43.0)	133 (39.0)	0.296.0
	Yes	176 (57.0)	208 (61.0)	
Tumour location	Right and transverse	108 (35.0)	139 (40.8)	0.041
	Left	64 (20.7)	81 (23.8)	
	Rectum	127 (41.1)	110 (32.3)	
	Total and subtotal	10 (3.2)	11 (3.2)	
Adjuvant chemotherapy	No	103 (33.3)	84 (24.6)	0.027
	Yes	206 (66.7)	257 (75.4)	
	Systemic inflammation			
mGPS	0	242 (78.3)	257 (75.4)	0.045
	1	35 (11.3)	28 (8.2)	
	2	32 (10.4)	56 (16.4)	
NLR	≤3	183 (59.2)	186 (54.5)	0.229
	>3	126 (40.8)	155 (45.5)	
NPS	0	273 (88.3)	295 (86.5)	0.738
	1	30 (9.7)	37 (10.9)	
	2	6 (1.9)	9 (2.6)	
	Body composition			
BMI (kg/m^2^)	<25	136 (44.0)	83 (24.3)	<0.001
	≥25	173 (56.0)	258 (75.7)	
High SFI	No	76 (24.6)	40 (11.7)	<0.001
	Yes	233 (75.4)	301 (88.3)	
Visceral obesity	No	126 (40.8)	51 (15.0)	<0.001
	Yes	183 (59.2)	290 (85.0)	
Sarcopenia				
Low SMI (Martin)	No	196 (63.4)	171 (50.1)	<0.001
	Yes	113 (36.6)	170 (49.9)	
Low SMI (Dolan BMI ≥ 25)	No	204 (66.0)	167 (49.0)	<0.001
	Yes	105 (34.0)	174 (51.0)	
Low SMI (Caan)	No	179 (57.9)	134 (39.3)	<0.001
	Yes	130 (42.1)	207 (60.7)	
Low SMI (Dolan BMI ≥ 30)	No	211 (68.3)	175 (51.3)	<0.001
	Yes	98 (31.7)	166 (48.7)	
Myosteatosis				
Low SMD (Martin)	No	233 (75.4)	25 (7.3)	<0.001
	Yes	76 (24.6)	316 (92.7)	
Low SMD (Dolan BMI ≥ 25)	No	303 (98.1)	40 (11.7)	<0.001
	Yes	6 (1.9)	301 (88.3)	
Low SMD (Dolan Male/Female)	No	284 (91.8)	20 (5.9)	<0.001
	Yes	25 (8.1)	321 (94.1)	

BMI, body mass index; mGPS, modified Glasgow prognostic score; NLR, neutrophil lymphocyte ratio; NPS, neutrophil‐platelet score; SFI, subcutaneous fat index; SMD, skeletal muscle density; SMI, skeletal muscle index.

**Table 5:** The relationship between mGPS, clinicopathological characteristic, and systemic inflammation in patients undergoing elective surgery for colorectal cancer (*n* = 650)

Characteristic		mGPS 0	mGPS 1 and 2 (*n* = 151)	*P*‐value
	Clinicopathological			
Age	≤65	185 (37.1)	49 (32.5)	0.410
	65–74	193 (38.7)	58 (38.4)	
	>74	121 (24.2)	44 (29.1)	
Sex	Female	228 (45.7)	68 (45.0)	0.887
	Male	271 (54.3)	83 (55.0)	
ASA score	1	120 (24.0)	21 (13.9)	0.036
	2	221 (44.3)	76 (50.3)	
	3	146 (29.3)	47 (31.1)	
	4	12 (2.4)	7 (4.6)	
Laparoscopic surgery	No	303 (60.7)	104 (68.9)	0.070
	Yes	196 (39.3)	47 (31.1)	
TNM	0	13 (2.6)	1 (0.7)	<0.001
	I	135 (27.1)	20 (13.2)	
	II	173 (34.7)	90 (59.6)	
	III	178 (35.7)	40 (26.5)	
Venous invasion	No	199 (39.9)	67 (44.4)	0.325
	Yes	300 (60.1)	84 (55.6)	
Tumour location	Right and transverse	175 (35.1)	72 (47.7)	0.014
	Left	112 (22.4)	33 (21.9)	
	Rectum	197 (39.5)	40 (26.5)	
	Total and subtotal	15 (3.0)	6 (4.0)	
Adjuvant chemotherapy	No	293 (66.9)	92 (68.7)	0.704
	Yes	206 (33.1)	59 (31.3)	
	Systemic inflammation			
NLR	≤3	308 (61.7)	61 (40.4)	<0.001
	>3	191 (38.3)	90 (59.6)	
NPS	0	459 (92.0)	109 (72.2)	<0.001
	1	38 (7.6)	29 (19.2)	
	2	2 (0.4)	13 (8.6)	
	Body composition			
BMI (kg/m^2^)	<25	156 (31.3)	63 (41.7)	0.017
	≥25	343 (68.7)	88 (58.3)	
High SFI	No	84 (16.8)	32 (21.2)	0.220
	Yes	415 (83.2)	119 (78.8)	
Visceral obesity	No	129 (25.9)	48 (31.8)	0.151
	Yes	370 (74.1)	103 (68.2)	
Low SMI (sarcopenia)				
SMI (Martin)	No	298 (59.7)	69 (45.7)	0.002
	Yes	201 (40.3)	82 (54.3)	
SMI (Dolan BMI ≥ 25)	No	299 (59.9)	72 (47.7)	0.008
	Yes	200 (40.1)	79 (52.3)	
SMI (Caan)	No	254 (50.9)	59 (39.1)	0.011
	Yes	245 (49.1)	92 (60.9)	
SMI (Dolan BMI ≥ 30)	No	309 (61.9)	77 (51.0)	0.017
	Yes	190 (38.1)	74 (49.0)	
Low SMD (myosteatosis)				
SMD (Martin)	No	214 (42.9)	44 (29.1)	0.002
	Yes	285(57.1)	107 (70.9)	
SMD (Dolan BMI ≥ 25)	No	274 (54.9)	69 (45.7)	0.047
	Yes	225 (45.1)	82 (54.3)	
SMD (Xiao)	No	242 (48.5)	67 (44.4)	0.374
	Yes	257 (51.5)	84 (55.6)	
SMD (Dolan Male/Female)	No	241 (48.3)	63 (41.7)	0.156
	Yes	258 (51.7)	88 (58.3)	

BMI, body mass index; mGPS, modified Glasgow prognostic score; NLR, neutrophil lymphocyte ratio; NPS, neutrophil‐platelet score; SFI, subcutaneous fat index; SMD, skeletal muscle density; SMI, skeletal muscle index.

**Figure 2:**
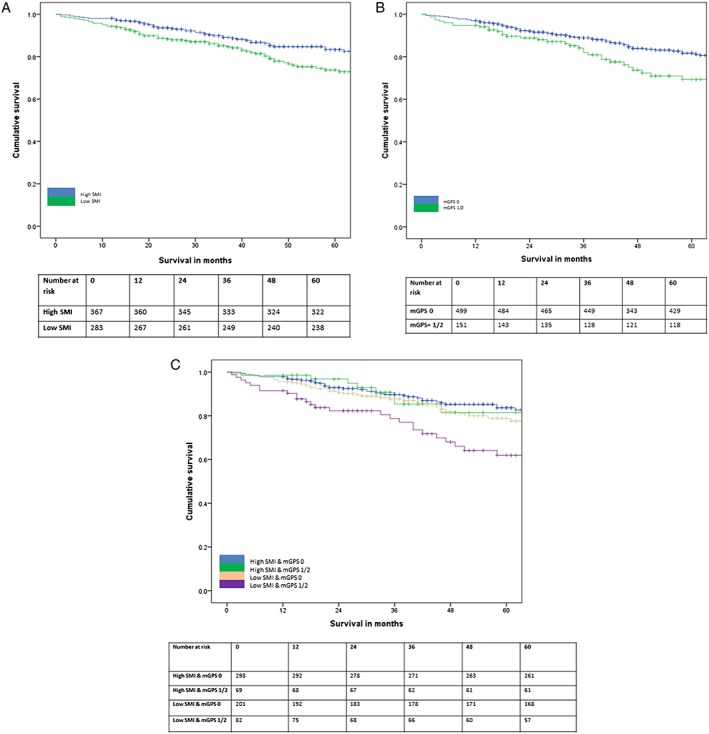
(A) The relationship between skeletal muscle index (SMI) (Martin) and overall survival (*n* = 650, *P* = 0.002). (B) The relationship between skeletal muscle density (SMD) (Xiao) and overall survival (*n* = 650, *P* = 0.019). (C) The relationship between modified Glasgow prognostic score (mGPS) and overall survival (*n* = 650, *P* = 0.010).

The relationship between SMI (Martin) high/low groups, SMD (Xiao) high/low groups, and mGPS high/low groups and overall survival is shown in *Figure* 2. Comparing SMI (Martin), SMD (Xiao), and mGPS, SMI (Martin) (HR 1.50, 95% CI 1.04–2.18, *P* = 0.031), SMD (Xiao) (HR 1.42, 95% CI 0.98–2.05, *P* = 0.061), and mGPS (HR 1.44, 95% CI 1.15–1.79, *P* = 0.001) were independently associated with overall survival (*Table* 6).

**Table 6:** The relationship between SMI, SMD, mGPS, sarcopenia, and overall survival in patients undergoing elective surgery for colorectal cancer (*n* = 650)

Independent, mutually adjusted association	HR (95% CI)	*P*‐value
All Patients *n* = 650		
mGPS	1.44 (1.15–1.79)	0.001
Low SMI (Martin)	1.50 (1.04–2.18)	0.031
Low SMD (Xiao)	1.42 (0.98–2.05)	0.061
mGPS 0 *n* = 499		
Low SMI (Martin)	1.48 (0.97–2.28)	0.071
Low SMD (Xiao)	1.50 (0.97–2.33)	0.068
mGPS 1/21 *n* = 151		
Low SMI (Martin)	2.02 (0.98–4.18)	0.058
Low SMD (Xiao)	1.30 (0.67–2.54)	0.438

CI, confidence interval; HR, hazard ratio; mGPS, modified Glasgow prognostic score; SMD, skeletal muscle density; SMI, skeletal muscle index.

In patients with an mGPS of 0, SMI (Martin) (HR 1.48, 95% CI 0.97–2.28, *P* = 0.071) and SMD (Xiao) (HR 1.50, 95% CI 0.97–2.33, *P* = 0.068) were weakly associated with overall survival (*Table* 6). In patients with an mGPS of 0, SMI (Martin) (HR 2.02, 95% CI 0.98–4.18, *P* = 0.058) was weakly associated with overall survival (*Table* 6).

Low SMI (Martin) was present in 40% of patients with an mGPS of 0. In contrast, low SMI (Martin) was present in 66% of patients with an mGPS of 2. Low SMD (Xiao) was present in 52% of patients with an mGPS of 0. In contrast, SMD (Xiao) was present in 64% of patients with an mGPS of 2. A combination of low SMI (Martin) and low SMD (Xiao) was present with an mGPS 0 in 23.4% of patients. In contrast, a combination of low SMI (Martin) and low SMD (Martin) was present with an mGPS 2 in 45.5% of patients.

## Discussion

The results of the present comprehensive study, in patients with CRC who were largely overweight, and using CT‐derived body composition analysis, showed that sarcopenia (SMI) and myosteatosis (SMD) were significantly associated with survival. Moreover, SMI and SMD were associated with the presence of a systemic inflammatory (in particular the mGPS) and had independent prognostic value. Therefore, the present results support the routine measurement of the SMI, SMD, and mGPS as part of the clinical and nutritional assessment in patients with cancer.^3^, ^23^, ^30^

Colorectal cancer has been extensively examined with reference to CT‐derived body composition, and most studies have reported that either SMI or SMD is associated with survival. In contrast, few studies have included a measurement of the systemic inflammatory response in their analysis. In those studies that included a white cell measure of the systemic inflammatory response such as NLR, SMI and SMD were reported to be independently associated with survival.^17^, ^22^ Irrespective, the systemic inflammatory response (however measured) is associated with lower SMI and SMD. These observations may have profound implications for the treatment of sarcopenia and myosteatosis in patients with CRC and, potentially, other common solid tumours.

Such cross‐sectional data cannot determine whether a low SMI or SMD results in the presence of systemic inflammation or whether the presence of systemic inflammation results in low SMI or SMD. From the present results, it is clear that a low SMI, SMD, or both can occur in the absence of systemic inflammation. However, the proportion of patients with a low SMI, SMD, or both are substantially greater in the presence of systemic inflammation. It may be that in those patients that simply improving dietary intake and activity will improve SMI and SMD. In contrast, in those patients with an mGPS 1/2, it may be that moderation of the systemic inflammatory response is required in addition to improve SMI and SMD.^15^ In order to better understand the nature of this relationship, it will be important to carry out longitudinal and intervention studies.

With reference to longitudinal studies, Wallengren *et al*. reported that, in 471 patients with advanced cancer, a C‐reactive protein > 10 mg/L had less muscle mass (using dual energy X‐ray absorptiometry) on study entry and lost muscle at an accelerated rate during follow‐up.^31^ Malietzis *et al*. reported that, in 856 patient with operable CRC, an NLR > 3 was associated with lower muscle mass (CT scan) over time.^32^ Both studies concluded that systemic inflammation was a risk factor for muscle loss and may be a useful marker of catabolic drive. However, the loss of muscle quality has yet to be examined in this relationship. Therefore, further longitudinal studies are required if the relationship between skeletal muscle mass and quality, the systemic inflammatory response and survival is to be further elucidated. To our knowledge, the above relationship has not been examined in interventional studies.

It was of interest that, in the present study, ~50% of patients had a low SMI or SMD. Compared with other cohorts of patients with early stage CRC treated with surgical resection, these figures appear high and similar to that reported in the terminal stage of the disease. Given that these percentages were similar using various thresholds of Dolan, Martin, Caan, and Xiao for patients in this cohort, this may suggest that there is a baseline level of poor muscle quantity and quality within this population. This is perhaps not surprising given the deprivation levels of patients referred to Glasgow Royal Infirmary. Indeed, in Glasgow, 190 000 or just under 32% of the city's population resides in the 10% of the most deprived areas of the UK (the so‐called Glasgow effect). This is associated with a poor diet and physical fitness and high levels of alcohol consumption and smoking, which would have a direct effect on both muscle quantity and quality. Indeed, when direct comparisons are made with functional testing such as the ASA scoring in the present and other reported studies, for example, in the present study, 33% of patients had an ASA score of ≥3 (severe systemic disease) compared with a recent combined study of 2100 UK and Canadian patients undergoing elective surgery for CRCs where 20% had an ASA score of ≥3.^33^ In addition, when the 763 UK‐based patients of this study were examined in isolation, 11% had an ASA score of ≥3.^17^ Therefore, it is clear that the present patient cohort had higher levels of co‐morbid disease and lower levels of physical function and this may account for, in part, the high percentage of patients with a low SMI and SMD.

Indeed, it was of interest that in the present study, ASA was significantly associated with SMD and not SMI. A similar relationship has recently been reported between SMD but not SMI and the Charleston co‐morbidity index.^26^ This confirms the clinical utility of SMD as there is increasing recognition that an increase in muscle mass is not necessarily associated with an increase in function.^34^, ^35^ It may be that an improvement in muscle quality rather than mass will result in an improvement in physical function.

Limitations of the present study include its retrospective nature and that only patients with an electronically available CT scan were included. However, the study population was relatively large, well documented in terms of clinicopathological characteristics and measures of the systemic inflammatory response and relatively mature follow‐up. Furthermore, different validated threshold values were applied to the CT body composition parameters.

In summary, the present study provides comprehensive evidence that both low skeletal muscle mass and quality has a significant relationship to the systemic inflammatory response and to survival in patients with operable CRC. This supports the incorporation of the SMI, SMD, and mGPS as part of the clinical and nutritional assessment in patients with cancer. This relationship also suggests potential therapeutic interventions.

## Conflict of interest

The authors declare that they have no conflict of interest.
